# Webster’s Dictionary

**Published:** 1857-11

**Authors:** 


### DICTIONARY.

An American Dictionary of the English Language, with an Introductory Dissertation on the Origin, History, and Connection of the Languages of Western Asia and Europe, with an Explanation of the Principles on which Languages are formed.

By Noah Webster, LL.D.

Revised and enlarged by Professor Goodrich, of Yale College, with Pronouncing Vocabularies of Scripture, Classical, and Geographical Names. 1857. Published by the Messrs. Merriam, of Springfield, Mass.: 1460 pages, crown quarto, unabridged, each page having three columns, containing three times the amount of any other English Dictionary compiled in this country, comprising over 12,000 Geographical Names, and recommended by many of the greatest minds of the nation, Webster, Benton, Cass, Winthrop, Cox, Beecher Stowe, Humphrey, and others, as the most complete, accurate, and reliable Dictionary of the Language.—So that in this country the sale of Webster's Dictionaries, for the last ten years, has been tenfold greater than all other English Dictionaries besides, while in England it has not one prominent and distinct competitor for public usage; and is of such popularity there, that Bohn, who owned the plates of Worcester's largest work, is said to have altered the title page, and mutilated the preface, and published the work, as compiled from the materials of Noah Webster, LL.D., by Joseph E. Worcester.

---

Assurance of certainly curing you of any malady, always comes from a man who neither understands his business nor himself.

---

An Absurdity.—To purchase wood by measure. Its heat-producing qualities are in proportion to its weight, if seasoned. When in Paris, our wood was furnished by the pound.

